# Remedy for Cancer

**Published:** 1868-03

**Authors:** 


*Remedy for Cancer.*—Dr. Hood, in a paper published in the *London Lancet* for January, reports a case of recovery from mammary cancer in an old woman of over eighty years of age. The remedy used by this ancient dame is of such a simple character, and the whole statement is made in such evident good faith, that we transcribe the concluding portion of his communication entire.

"Although the diseased mass had separated itself from this lady's person, there still remained the peculiar odor common to external sloughing cancer. I inquired how it was she had resorted to this remedy, and she gave me the following history almost *totidem verbis*:—

"Some years ago a gentleman living near the town in which she resided had a tumor in his cheek as large as a small orange. It was about the time that the late Sir Astley Cooper gave up practice in London and came to reside at Hemmel Hempstead. This gentleman thought that he could not do better than go and consult Sir Astley about this tumor. Sir Astley saw it, and told him that it was a cancerous tumor; and strongly advised him to submit to its removal. This the gentleman objected to have done; when Sir Astley told him that unless he did he would not live beyond six months. On the following Tuesday, being market day, this gentleman was riding through the town, when an old woman accosted him by saying: 'I hope Sir you will pardon me; but, seeing your face tied up, may I ask what is the matter with you?' His reply was: 'My good woman, I have a cancer in my cheek; and Sir Ashley Cooper says I shall not live six months.' She rejoined: 'If you will try an old woman's remedy, I am sure it will cure it.' This the gentleman agreed to do; and in less than six months the tumor disappeared, and he got perfectly well."

This lady knew all the parties, and it was the success which attended the remedy in this gentleman's case which induced her to employ it in her own. The following is a verbatim copy she gave me of the directions for using the oyster-shell powder:—

"*For a Cancer.*

"Bake a quantity, say half a peck, of oyster-shells for three nights in a slow oven. Then scrape out the *small white* part of the shell, powder finely, and take as much as will lie on a shilling once or twice a day in a little warm water or tea. If that affects the system too much leave off a day or two and commence again.

"Should an ointment be thought desirable, mix the powder in cream, lard, or quite fresh butter without any salt in it, and apply it.

"This treatment generally requires perseverance for three or four months before its effects are seen.

"The shells to be used are those which are *concave.*"

This lady lived for two years after my first visit to her, and ultimately died in en epileptic convulsion, when she was apparently in her usual health. The wound resulting from the separation of the cancerous breast never entirely healed, but she never complained of any discomfort from it.

I have been in possession of these facts for some years, but should not have made them public, owing to the difficulty I felt in explaining the *modus operandi* of the apparently simple remedy of powdered oyster-shell in so formidable a disease as cancer—notwithstanding that I had seen in the case of the lady recorded so remarkable a result from the use of the remedy, and having every reason to believe, from her known truthfulness the account she gave me of the gentleman who had experienced such decided benefit from it,—had it not been for a conversation I had recently with Mr. Spencer Wells, to whom I related the substance of the foregoing statement. He informed me that he attributed the efficacy of the remedy entirely to the lime contained in the powder. He told me that since he had read Dr. M'Clintock's observations some years ago on the influence of the chloride of calcium in checking hæmorrhage in patients who had fibroid tumours of the uterus, he had used lime largely in the treatment of these and other tumours; and he had become convinced that an atrophy and calcification of fibroid tumours, resembling the spontaneous change or degeneration not unfrequently observed in such tumors, was often produced or hastened by the use of lime. And he added that he had reason to believe the change commenced in the coats of the arteries supplying the tumors with blood; that these coats underwent first an atheromatous, afterwards a calcareous degeneration—in either case with a diminution of the calibre of the vessel and a lessened supply of blood. If the lime were too long continued, he believed that all the arteries in the body, not those in the tumour only, began to degenerate; the first evidence being the formation of an arcus senilis around the cornea.

The explanation appeared so philiosophical, that I felt I ought no longer to observe silence on a subject of so much importance, but to submit the knowledge of it to those whose opportunities of seeing diseases of a cancerous nature are greater than mine, and who might feel disposed to give the remedy a trial; as it is one that is not likely to do harm and *may* in some instances do good.

If there be any force in the reasoning of Mr. Spencer Wells on the facts which he has observed, I cannot see why the nutrition of malignant tumors should not be as readily affected by inducing atheromatous or calcareous degeneration of the vessels which supply them with blood, as the nutrition of innocent tumours would appear to be.

Lower Seymour-street, Portman-square, Sept., 1867.

